# Nanostructuring and Underscreening of Bisalt Electrolytes with Dual-Anion Effects: Insights from Small-Angle Scattering Prepeak Analysis

**DOI:** 10.1021/acs.jpcb.6c01734

**Published:** 2026-07-27

**Authors:** Hsiu-Wen Wang, Emily T. Nienhuis, Ashley R. Kennedy, Sebastian T. Mergelsberg, Valerie Munoz, Lawrence M. Anovitz, Gergely Nagy, Jacob G. Reynolds, Jaehun Chun, Gregory K. Schenter, Carolyn I. Pearce

**Affiliations:** † Chemical Sciences Division, 6146 Oak Ridge National Laboratory, Oak Ridge, Tennessee 37831, United States; ‡ Neutron Scattering Division, 6146 Oak Ridge National Laboratory, Oak Ridge, Tennessee 37831, United States; § 6865 Pacific Northwest National Laboratory, Richland, Washington 99352, United States; ∥ 384929 Washington River Protection Solutions, LLC, Richland, Washington 99352, United States

## Abstract

Using small-angle neutron scattering, we studied the nanostructure of a prototypical D­(H)-bonded network electrolyte, alkaline sodium aluminate bisalt, at concentrations up to solute volume fraction of ∼0.5. Analysis of the structure factor prepeak at 0.1 < *Q* < 1.3 Å^–1^ showed that its evolution is associated with nanoscopic species distribution in water–anion network, with differences in characteristic distance *d* between OD^–^ and Al­(OD)_4_^–^ anions related to their distinct sizes and interactions. When the solute volume fraction approached 0.5, a common/maximum value of ∼4.5 Å was found for the correlation length ξ that characterizes the electrostatic force in concentrated electrolyte solution before precipitation occurred. This implies that the solutions’ morphology and behavior at high concentrations may be governed by geometric factors, rather than the chemistry of the specific anion. Furthermore, the scaling of ξ with concentration yielded an exponent of 1.25(1), suggesting that ξ is limited to a few Å. By combining the current observations of structural heterogeneity at the nanoscale, with dynamic heterogeneity at the microscopic scale from our previous quasi-elastic neutron scattering study, we have established a structural origin of local “caging” and restricted structural relaxation processes. These local solvent–solute interactions not only control dynamics heterogeneity in concentrated electrolytes but are also responsible for crystallization processes in industrial setting, such as aluminum production and radioactive waste treatment.

## Graphical abstract

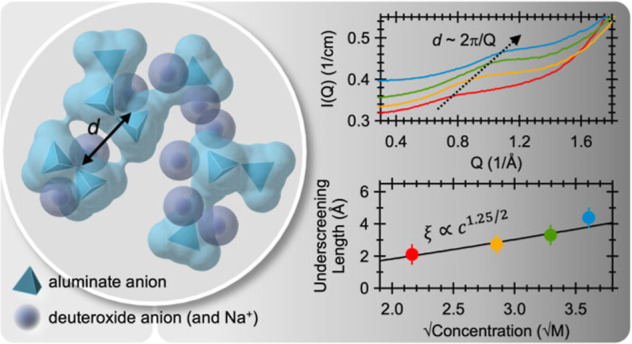

## Introduction

The idea of water-in-salt electrolytes (WISEs) originates from the condition when the salt outnumbers the solvent in a given binary system by both weight and volume.^1^ Li-based WISEs for batteries and supercapacitor applications stand out as one classic example, in which mixtures of Li^+^ with an organic polyatomic anion, such as trifluoromethanesulfonate (Tf^–^), bis­(fluorosulfonyl)­imide (FSI^–^), bis­(trifluoromethanesulfonyl)­imide (TFSI^–^), and bis­(pentafluoroethanesulfonyl)­imide (BETI^–^), at concentrations up to double digits in molality (m, moles per kilogram of solvent), represent such ion-rich networks that disrupt water’s hydrogen-bonding (H-bonding) environments.^1–4^ This concept was extended to the highly water-soluble inorganic salt or bisalt electrolytes at concentrations when the number of water molecules available to solvate each ion is far below the solvation numbers established in dilute concentrations (< ∼1.0 m).^5,6^ In the hydration number definition, high solubility, low water-to-ion ratio, and prevalence of ionic aggregation are the common features,^7,8^ regardless of whether the solutes actually exceed the solvent by weight or volume. Examples for simple salt electrolytes are lithium chloride (LiCl, solubility at 25 °C = 19.9 m,^9^ water-to-cation ratio = 2.8), sodium perchlorate (NaClO_4_, 16.7 m,^9^ 3.3), potassium hydroxide (KOH, 21.5 m,^9^ 2.6), rubidium carbonate (Rb_2_CO_3_, 10.5 m,^9^ 0.5), and cesium fluoride (CsF, 34.8 m,^9^ 1.6). Bisalt electrolytes that show enhanced solubilities (or metastability) with multiple-ion effects include many alkali nitrate solutions,^10^ e.g., sodium nitrate-rubidium nitrate (NaNO_3_–RbNO_3_–H_2_O), alkaline aluminate solutions,^11,12^ e.g., sodium aluminate-sodium hydroxide (NaAl­(OH)_4_–NaOH–H_2_O), and metal chloride solutions,^13^ e.g., zinc chloride-lithium chloride (ZnCl_2_–LiCl–H_2_O). Regardless of the definition selected, WISEs are characterized by low water activity, a balance between ion solvation and ion pairing, and an enthalpy–entropy compensation that governs the distribution of species and heterogeneity at the nanoscale.

The network structure of WISEs has been the subject of multiple investigations covering various spatiotemporal regimes. Investigations have related the extent of ion aggregation to the formation and reactivity of associated species/complexes, leading to multiple nonclassical nucleation pathways and, in some cases, phase selections, e.g., amorphous intermediates have been observed prior to bulk crystal precipitation.^6,11,14^ It has also been shown that complex water–ion molecular networks form from isolated aqueous ions and result in high viscosities that can fluctuate unpredictably between liquid and near-solid, glass-like states.^5^ Studies of aqueous battery performance have revealed that transport properties, such as viscosity, diffusion, and conductivity, are linked to ionic packing arrangements and the available pathways for ion movement.^7,13,15,16^

In the neutron or X-ray scattering experiments, the near-neighbor pairwise correlations in aqueous electrolytes contribute to the first sharp diffraction peak at wavevector (*Q*) around 1.5–3.5 Å^–1^. The position and intensity of this peak provide information on the local molecular structure (< ∼5 Å) of hydration spheres, ionic complexes, and the H-bonded network.^17,18^ At smaller scattering *Q* values between 0.1 and 1 Å^–1^, a prepeak hump or simply a rise in scattering intensity (often following a *Q* ^–*n*^ power law) may appear. This is often ascribed to intermediate-range ordering.^13,19–22^ The intensity behaviors in this small-*Q* region display strong concentration dependencies and salt/solvent identity effects.^21^ For instance, small-angle X-ray scattering (SAXS) profiles of KNO_3_ and RbNO_3_ aqueous solutions close to their solubility limits (3.8 and 4.4 m, respectively, at 25 °C) display only simple power law intensity behavior, together with a “knee” where the curve bends into a plateau at smaller *Q*.^23^ Such feature is associated with nanoemulsion-like behavior of salt-in-water systems in which electron density fluctuations are caused by the thermal motion of ions and water molecules. The location of the knee, usually at *Q* ∼ 0.4 Å^–1^ (2π/*Q* ≈ 16 Å), roughly corresponds to the spatial extent (correlation length) of electron-dense heterogeneities and relates to intermediate-range ion–ion and ion–water interactions.^23^ In contrast, SAXS profiles of divalent cation chloride salt solutions (MgCl_2_, CaCl_2_, SrCl_2_, BaCl_2_) or trivalent cation chloride salt solutions (ErCl_3_) have a clear prepeak signal.^19,21^ Molecular dynamic simulations suggest that the SAXS prepeak is a collective structural correlation effect, meaning that some intermediate-range atom–atom correlations are positive and some are negative, and the scattering prepeak depends on how these structure factors interfere, both destructively and constructively.^24^ The SAXS prepeak in chloride salt solutions mainly results from the intermolecular cation···O_w_ (water-oxygen), O_w_···O_w_, and cation···cation correlations between two different solvated cationic species, that present as one molecular entity separated by some intermediate distance (∼8–11 Å).^19^ Hence, the position of the maximum intensity of the prepeak directly relates to the length scale of the structural arrangement at the nanoscale, sometimes termed supra-arrangements.^19^ This has been shown in SAXS profiles of LiTFSI aqueous electrolytes (the most studied WISEs) in which the prepeak shifts from *Q* ∼ 0.3 Å^–1^ to 0.5 Å^–1^ (2π/*Q* ≈ 20.9 Å to 12.5 Å) as the concentration increases from 1 to 20 m.^16^ Since the X-ray scattering power of Li^+^ is much weaker than TFSI^–^, SAXS measurements of LiTFSI aqueous electrolytes mainly probe the nanometer scale arrangement of TFSI^–^ anions. The observed shift in the prepeak thus indicates that the characteristic distance *d* (≈ 2π/*Q*) between solvated TFSI^–^ anions becomes shorter with increasing concentration and eventually forms a percolating anionic network with trapped water molecules and Li^+^ when the concentration reaches ∼20 m.^16,25^

Here, we investigate the nanoscale structure of alkaline sodium aluminate bisalt electrolytes (NaAl­(OH)_4_–NaOH–H_2_O) by varying both total salt concentration and anion ratio to determine the factors that affect the solutions’ metastability. This solution system is central to the Bayer process in the alumina industry^26,27^ and is also important for radioactive waste stored at the Department of Energy (DOE)’s legacy nuclear sites, e.g., Hanford,^28,29^ where chemical conditions must be tuned to stabilize the solutions and prevent precipitation during processing.^30,31^ It has been argued that the two primarily anions, aluminate (Al­(OH)_4_^–^) and hydroxide (OH^–^), in the NaAl­(OH)_4_–NaOH–H_2_O solution system actively control ionic complex formation and alter nucleation behavior.^12^ The presence of excess charge-balancing OH^–^ anions is critical for aluminate polymerization as they facilitate H-bonding and promote proton transfer.^12,32^ Equally important, the tetrahedral geometry of the aluminate species (the Al­(OH)_4_^–^ monomer, the μ_2_-oxo Al_2_O­(OH)_6_^2–^ dimer, or larger oligomers with the μ_2_-oxo linkage) and water’s tetrahedral H-bonding, create network structures in which various tetrahedral motifs can be packed and connected.^33^ Under water-in-salt conditions (i.e., a deficiency of water molecules), this tetrahedral network may be responsible for the prolonged metastability,^12^ frustrated crystallization behavior (at room temperature),^6^ and good glass-forming ability^34^ (upon supercooling to ∼200–150 K) in the NaAl­(OH)_4_–NaOH–H_2_O solutions.

Small-angle neutron scattering (SANS) profiles of deuterated NaAl­(OD)_4_–NaOD–D_2_O solutions were collected to determine the nanoscale structural heterogeneity and species distribution of D_2_O and OD-containing moieties (OD^–^ and aluminate species). Deuteration was used to lower the inelastic contribution due to the smaller incoherent scattering of D relative to H. The effect of anion size and ratio (OD^–^ vs Al­(OD)_4_^–^) and the impact of total salt concentration, from water-in-salt to salt-in-water conditions, were explored. Furthermore, we relate these observations of nanoscale structural heterogeneity to our previous quasi-elastic neutron scattering (QENS) and proton nuclear magnetic resonance spectroscopy (^1^H NMR)^5,6^ studies, showing the structural origin of microscopic dynamic heterogeneity for the greater local and confined motions of ionic complexes in the system.

## Experimental Section

### Concentration Series of Alkaline Sodium Aluminate Solutions

Sample preparation was performed in a nitrogen (N_2_)-filled glovebox. The N_2_ environment protects against carbon dioxide (CO_2_) capture, which is significant in concentrated NaOD solutions, and prevents H/D isotope exchange. Deuterated sodium aluminate solutions were generated by quantitative dissolution of degreased Al wire (99.999% grade) in 40 wt % stock NaOD solutions (in D_2_O; Sigma-Aldrich, 372072, 99.5 atom % D), with D_2_O water (Cambridge Isotope Laboratories, inc., DLM-6-S-50, 99.96 atom % D) addition or removal (by evaporation inside the glovebox over a hot plate) as needed to achieve the desired [Na^+^]_tot_ and [Al^3+^]_tot_ concentrations. All deuterated chemicals were used as received. The dissolution of Al wire can be expressed as *x*Al_(s)_ + *y*NaOD_(aq)_ + 3*x*D_2_O_(aq)_ → *x*Al­(OD)_4_^–^ + *y*Na^+^ + (*y*–*x*)­OD^–^ + (3*x*/2)­D_2(g)_, where the predominant form of Al^3+^ is the aluminate anion, Al­(OD)_4_^–^. The variable denoted (*y*–*x*) defines the amount of excess OD^–^ that is not consumed by Al­(OD)_4_^–^ formation. This is subject to change with the formation of dimeric or polynuclear aluminate complexes. It is worth noting that only a small range of aqueous aluminate species, including monomer and dimers, can be observed in solution by infrared or Raman spectroscopy together with theoretical mode assignments.^35^ The existence of higher order oligomeric species remains difficult to identify using both spectroscopic and scattering techniques, because they bear similar motifs and linkages as in monomeric and dimeric species and the unknown ion association effects that could lead to the overlapping bands and uncertainties in distinguishing these structural motifs.^11,12^ However, under high concentrations, where competition for water molecules occurs, there is a thermodynamic driving force for ionic cluster formation, consisting of a deuterium (hydrogen)-bonded polyhedral network.

On the basis of the above expression, two solution series with a Na^+^/Al^3+^ mole ratio of 2 (R2) and 3 (R3) were prepared, respectively, with the final concentrations given in Table 1. Note that the amount of Al wire dissolved is calculated on the basis of 55.506 mol of D_2_O with the aluminate monomer speciation balance assumed, i.e., additional D_2_O molecules formed via condensation of Al­(OD)_4_^–^ into Al_2_O­(OD)_6_^2–^ were ignored. Based on the concentrations listed in Table 1, R2 solutions can be viewed as having one-half (50 mol %) of the OD^–^ replaced by Al­(OD)_4_^–^ and R3 solutions as one-third (33.3 mol %) of OD^–^ being replaced by Al­(OD)_4_^–^. Figure 1 illustrates the relation of each solution series to the water-in-salt regime, where there is insufficient D_2_O to fully solvate all ions in the system, necessitating shared hydration shells and ionic aggregation.

**Table 1:** Composition (Molality), Density (g/cm^3^; Measured vs Estimated), and Calculated Solute Volume Fractions ϕ_solute_ (from Each Density Data) of the Deuterated Alkaline Sodium Aluminate Bisalt Electrolytes

	[Na^+^]_tot_ (m)^a^	NaOD (m)^a^	NaAl(OD)_4_ (m)^a^	density (g/cm^3^) measured/estimated	ϕ_solute_ calculated based on measured or estimated density
R2	5.022	2.517	2.505	1.356/1.332	0.11/0.12
	10.062	5.043	5.020	1.509/1.471	0.22/0.23
	20.032	10.039	9.993	1.683/1.625	0.39/0.40
R3	5.192	3.460	1.732	1.360/1.318	0.07/0.09
	10.043	6.693	3.349	1.509/1.453	0.16/0.19
	14.936	9.955	4.981	1.608/1.544	0.25/0.27
	19.960	13.303	6.657	1.675/1.607	0.32/0.35
^b^	25.159	16.768	8.391	------/1.654	-----/0.41
^b^	30.133	20.083	10.050	------/1.689	-----/0.46

a Calculations were performed consistently on the basis of 55.506 mol of D_2_O. m = molality = (moles of solute)/(1.1102 kg of D_2_O) = (moles of solute)/(55.506 mol of D_2_O). Since all materials are weighed based on their mass, the three decimal place values in concentration are from a three-decimal point analytical balance with 0.001 g readability.

b Crystallization was observed in the solution during measurements.

**Figure 1:**
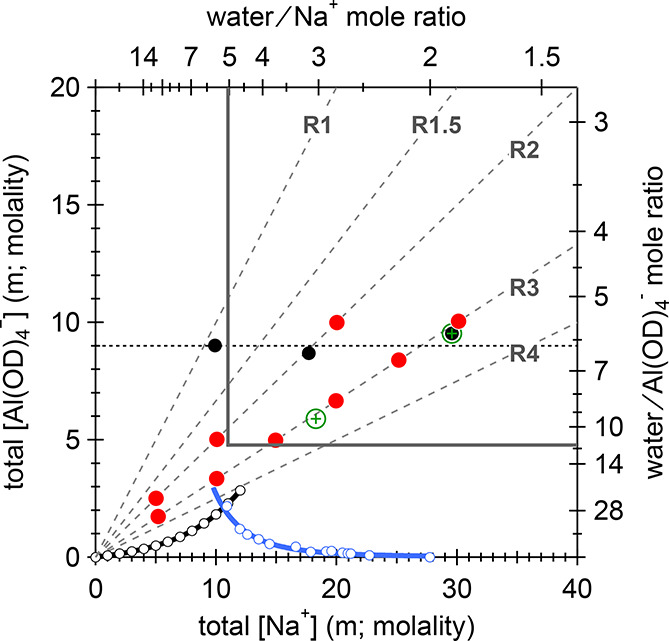
Chemical stability diagram of alkaline sodium aluminate bisalt electrolytes at 25–30 °C. Gibbsite and sodium aluminate salt solubility curves are shown by the thick black and blue lines, respectively.^36–38^ The maximum solubility is near 10 m [Na^+^]_tot_ and 2 m [Al^3+^]_tot_. Solution compositions for SANS measurements in this study are shown in solid red spheres, for the previous neutron total scattering study^12^ are in solid black spheres (following ∼9 m [Al^3+^]_tot_ concentration line), and for protiated samples measured in the previous QENS study^5,6^ are in open/cross green circles. The dashed diagonal lines follow constant Na^+^/Al^3+^ mole ratios of 1–4 (R1-R4). Solutions falling within the outline of the gray square (at the top-right) do not have enough water molecules to fully solvate all solute species and are considered water-in-salt solutions. Solubility curves were reproduced from Wang et al.^11^ Copyright 2022 American Chemical Society.

The two highest concentration samples (denoted with label-b in Table 1) showed visible crystallization during the SANS measurements. In our previous neutron total scattering-/radial distribution function (TS/RDF) analysis^12^ (on deuterated samples) and QENS measurements^5,6^ (on protiated samples), similar solutions were stable in a supersaturated state for extended periods of time. For these previous works, the solutions were prepared on-site, whereas in the current study, solutions were prepared offsite and shipped for analysis, therefore, it is possible that temperature fluctuations during shipment could have influenced solution stability. SANS results for the solutions with precipitates are shown for a qualitative comparison only.

### Solution Density and Solute Volume Fraction Estimation

The solution densities at 25 °C reported in Table 1 and shown in Figure S1a (Supporting Information) are based on density data in Radnai et al.,^18^ Sipos et al.,^39^ and Li et al.,^40^ using linear regression to estimate densities for the solutions studied (see Supporting Text-1 for details). As in our previous neutron TS/RDF work,^12^ the atomic weights of the different isotopes involved were considered in these estimations. To validate the estimated density, the density of select solution samples (note: not from the same synthesis batch) were measured experimentally using a Mettler Toledo Easy D40 density meter at 20 °C. Approximately 1 mL of sample was used in the measurement. Water was used to verify the calibration. These measured solution densities are also listed in Table 1. The solute volume fraction (ϕ_solute_) was calculated assuming that the solution is a mixture of two phases: the solute [*x*NaAl­(OD)_4_ + (*y*–*x*)­NaOD] phase in 55.506 mol of D_2_O solvent phase. Assuming the mixing volumes of the two components behave ideally, the apparent molar volume of the solution (*V*_m_; cm^3^/mol) will follow a linear trend as $V_{m}=\left(1-X_{solute}\right)V_{D_{2}O}+X_{solute}V_{solute}$ , where $V_{D_{2}O}$ and *V*_solute_ are the apparent molar volume of the water and solute phases, respectively, and *X*_solute_ = *y*/(*y* + 55.506) is the mole fraction of the solute phase. Note that *V*_m_ can be calculated from the solution density, and $V_{D_{2}O}=18.016\;\left({cm}^{3}/mol\right)$ . Thus, the solute volume fraction (ϕ_solute_) can be obtained as ϕ_solute_ = (*X*_solute_ *V*_solute_)/*V*_m_. Calculations of ϕ_solute_ on the basis of measured vs estimated densities are given in Table 1. In reality, the two-phase components are not composed of pure water and pure solute species (e.g., some Na^+^ and OD^–^ would partition between both phases), and aluminate can undergo polymerization, complicating the actual composition of the phase domains but, in our approximations, ϕ_solute_ is used as an estimate of a “domain” volume fraction to facilitate SANS data analysis. Figure S1b,c shows the approximated *V*_solute_ (cm^3^/mol) and ϕ_solute_ values as a function of the [Na^+^]_tot_ concentrations.

### Small-Angle Neutron Scattering Measurement

SANS measurements were carried out on the EQ-SANS instrument at the Spallation Neutron Source at Oak Ridge National Laboratory.^41,42^ The solution samples were loaded in quartz banjo cells with 2 mm flight paths. Measurements were performed at room temperature, with the neutron wavelength and sample-to-detector distance varied in three configurations: (i) a wavelength band of 2.5–6.4 Å at 2.5 m sample-to-detector distance, (ii) a wavelength band of 1–5.2 Å at 1.3 m sample-to-detector distance, and (iii) a wavelength band of 0.5–4.7 Å at 1.3 m sample-to-detector distance. The results were reduced after correcting for detector sensitivity and transmission, and subtracting background scatterings. The scattering intensities were then azimuthally averaged to obtain 1D scattering patterns. Together, scattering wavevectors in the range of 0.02 < *Q* < 4.8 Å^–1^ were covered. The data were converted to absolute scale intensities (cm^–1^) using a fitting to a porous silica standard sample in the 0.01–0.03 Å^–1^ *Q*-range.^43^

## Results and Discussion

### Concentration Dependence of the Bisalt Electrolyte Nanostructure

The SANS profiles with increasing total salt concentrations at room temperature are shown in Figure 2. Regardless of the anion mole ratio (R2 vs R3), as the concentration (solute volume fraction ϕ_solute_) increases, the structure factor primary peak shifts from *Q* of ∼ 2.0 Å^–1^ (in bulk D_2_O water; ϕ_solute_ = 0) to 2.4 Å^–1^ at ϕ_solute_ = 0.46, and the prepeak shifts from *Q* of ∼ 0.85 Å^–1^ to 1.11 Å^–1^ as ϕ_solute_ increases from 0.09 to 0.40 (Figure 2a,b). The primary peak is typically defined as the first sharp diffraction peak for bulk water and most aqueous electrolytes and is attributed to the extended D/H-bonded water network.^18^ The disrupting effect of high salt concentration on water’s tetrahedral network is evident by the shifting of this peak (Figure 2b). For instance, the position of the second-shell O_w_···O_w_ peak moves from ∼4.3 Å (in pure water) to ∼3.2 Å with increasing salt concentration, in a manner analogous to pure water under pressure, implying a more compact, high-density water structure.^44^ Because the structure factor is a sum of interfering waves, each originating from different local (<∼5 Å) molecular structural contributions, other structural factors, such as ion solvation and ion pairing, may also result in peak shifting. For the prepeak (Figure 2a), SANS probes the average structure of the neutron-illuminated objects (molecular motifs) in the solution. As described in our previous neutron TS/RDF work,^12^ three types of molecular motifs involving aluminate-D_2_O, OD^–^-D_2_O, and D_2_O–D_2_O correlations contribute most strongly to the measured intensities (see Table S1 and Supporting Text-2 for details). Thus, the largest scattering length density contrast is likely between the solute (mainly anions) and solvent (D_2_O) components. Accordingly, the intensity of the prepeak increases with ϕ_solute_, together with the observed change with concentration (Figure 2a), suggest that the structure factors between the solute and water domains increase, and the average distances between solute-rich nanoscale domains (shown as the prepeak) decrease, potentially reflecting aluminate polymerization.

**Figure 2:**
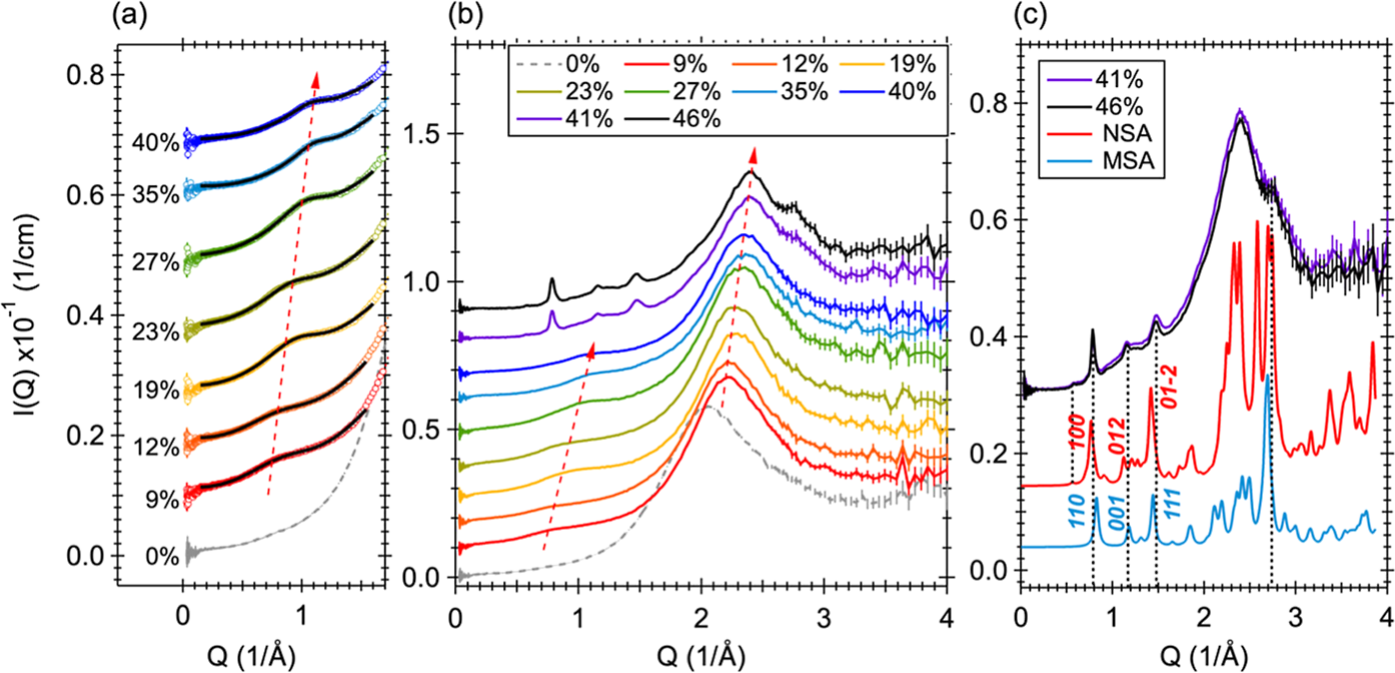
SANS profiles of alkaline sodium aluminate bisalt electrolytes (NaAl­(OD)_4_-NaOD-D_2_O) at different solute volume fractions (ϕ_solute_; in percentages). The data are offset for clarity. (a) The prepeak signal is emphasized and the solid black lines represent curve fitting with a Teubner–Strey model. (b) Both the prepeak and the primary peak are shown and marked by red dashed lines for clarity. (c) The appearance of Bragg reflections in the two highest concentration samples. Simulated neutron diffraction patterns of monosodium aluminate hydrate (MSA; blue) and nonasodium aluminate hydrate (NSA; red), using crystal structure data of Kaduk and Pei^45^ and Weinberger et al.,^46^ respectively, are presented as a guide to the eye.

Bragg diffraction peaks that emerged in the two highest concentration samples (Figure 2c; ϕ_solute_ = 0.41 and 0.46) are consistent with those calculated for crystalline sodium aluminate structures (Figure 2c), including monosodium aluminate hydrate^45^ (MSA: Na_2_[Al_2_O_3_(OD)_2_]·1.5D_2_O) and/or nonasodium aluminate hydrate^46^ (NSA: Na_9_[Al­(OD)_6_]_2_(OD)_3_·6D_2_O). Both MSA and NSA salt crystals are the solubility-controlling phases when the Na^+^/Al^3+^ mole ratios are greater than ∼1 and the [Na^+^]_tot_ is above ∼10 m (Figure 1). Although the prepeak is often associated with some underlying supra-molecular structure, in Figure 2, it appears as a broad hump that cannot be directly assigned the low-index atomic planes expected for MSA (e.g., the 001 reflection) or NSA (e.g., the 012 reflection) crystals.

### Teubner–Strey Bicontinuous Model Fit

Quantitative analysis of the SANS profiles was performed using the two-phase bicontinuous random distribution model proposed by Teubner and Strey.^47^ The Teubner–Strey (TS) model is widely applied to scattering data from aqueous electrolytes that show prepeak behavior.^13,16,19,21^ This model provides a good empirical description of a disordered and sponge-like morphology and incorporates two key constraints expected for nanosegregated liquids: (i) the alternating arrangement of the two phases, with the quasi-periodic repeat distance *d*, and (ii) the loss of long-range order, depicted by the correlation length ξ (i.e., the length beyond which the correlation dies out).^16,47^ The model assumes a pair correlation function of the form

1 $$\gamma \left(r\right)=\frac{d}{2\pi r}\exp\left(-\frac{r}{\xi }\right)\sin\left(\frac{2\pi r}{d}\right)$$

This structure factor can be incorporated into the macroscopic scattering cross section expression to generate a SANS *I*(*Q*) intensity of the form

2 $$I\left(Q\right)=\left(8\pi \phi \Delta \rho^{2}\frac{1}{\xi }\right)\times \left[\frac{1}{\left(\frac{a_{2}}{c_{2}}\right)+\left(\frac{c_{1}}{c_{2}}\right)Q^{2}+Q^{4}}\right]+background$$

where ϕ is volume fraction of a phase (in our approximation ϕ ≈ ϕ_solute_), Δρ^2^ is the square of the difference in the scattering length density between the two phases, and *a*_2_, *c*_1_, and *c*_2_ are order parameters (fit variables), relating to *d* and ξ as

3 $$d=2\pi {\left[\frac{1}{2}{\left(\frac{a_{2}}{c_{2}}\right)}^{1/2}-\frac{1}{4}\left(\frac{c_{1}}{c_{2}}\right)\right]}^{-1/2}$$

4 $$\xi ={\left[\frac{1}{2}{\left(\frac{a_{2}}{c_{2}}\right)}^{1/2}+\frac{1}{4}\left(\frac{c_{1}}{c_{2}}\right)\right]}^{-1/2}$$

Note that the scattering length density of each phase cannot be calculated, because it depends on the knowledge of actual partition of ions between the solute-rich and water-rich phase domains. A similar situation occurs with ϕ, but as mentioned above, ϕ is approximated as ϕ_solute_. In the fitting, these two terms were considered as a part of empirical proportionality factor (see Figure S2 for the fit function description). In eq 2, the high-*Q* intensity decays as *Q* ^–4^, which is Porod-limit behavior for phase domains delimited by a sharp, smooth interface. Thus, the surface to volume ratio (*S*/*V*) of the interface between two phases may be inferred from *I*(*Q* → ∞) as

5 $$\frac{S}{V}=\frac{4\phi \left(1-\phi \right)}{\xi }$$

The pair correlation function in eq 1 is based on liquid-state theory,^21,48^ which predicts that, as the salt concentration increases, there comes a point called a Kirkwood transition, where the asymptotic decay of the ion–ion (charge–charge) correlation function transits from purely exponential decay γ­(*r*) ∝ exp­(−*a*_0_ *r*) to damped oscillatory behavior γ­(*r*) ∝ exp­(−*a*_0_ *r*) cos­(*q*_0_ *r* – δ)/*r*.^48,49^ Accordingly, *a*_0_ = 1/ξ is the inverse of the decay length (electrostatic screening length), *q*_0_ = 2π/*d* is the oscillation frequency of periodicity at distance *d*, and δ is the phase shift. The quantitative connection between the prepeak in the SANS/SAXS profile and the collective effects in concentrated electrolytes can, therefore, be obtained through the fitting descriptors ξ (1/*a*_0_) and *d* (2π/*q*_0_).

The results of fitting the prepeak in the range of 0.16 ≤ *Q* ≤ 1.6 Å^–1^ with the TS model are displayed in Figure 2a (see also Figure S2 for fitting examples), and the parameters (*d*, ξ, and *S*/*V*) obtained from the fitting are shown in Figure 3 (see Table S2 for tabulated values). The shift in the prepeak maximum to higher *Q* values defines changes in the periodicity distance *d*, which is directly related to the length scales between scattering inhomogeneities in the solutions. Since the highest ϕ_solute_ value is less than 0.5, the two-phase solution morphology can be considered as solute-rich nanodomains (composed of a few anions) in a water-rich matrix. The decreasing trend observed for *d* (from ∼7.5 to 5.5 Å) in both solution series (Figure 3a) indicates that solute-rich nanodomains get closer to each other as the concentration increases. This solute clustering behavior may facilitate aluminate polymerization reactions and formation of prenucleation species. The substitution of Al­(OD)_4_^–^ for OD^–^ in R2 solutions (∼1.5*x* greater aluminate concentrations than in R3 solutions of the same [Na^+^]_tot_; Table 1) results in consistently larger *d* values than in the R3 solutions (Figure 3a). This likely reflects anion size effects, i.e., the Al­(OD)_4_^–^ monomers (or oligomers) are larger than OD^–^ and, consequently, a larger domain size is expected. The observed feature in *d* may also relate to the participation (chemical interaction) of Na^+^ and OD^–^ ions between the water-rich and aluminate-rich domains. However, the exact molecular origin remains to be determined, especially the role of OD^–^ in facilitating aluminate polymerization and anionic network formation.

**Figure 3:**
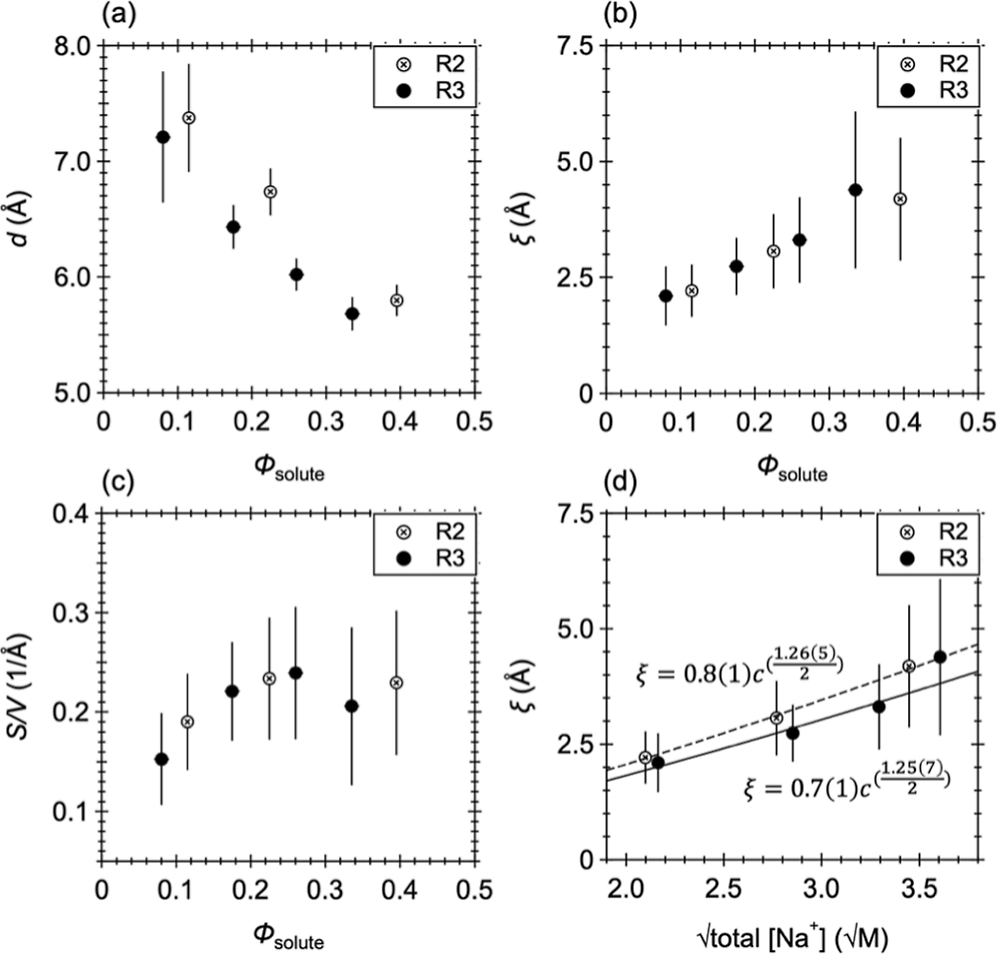
Evolution of (a) the periodicity distance *d*, (b) the correlation length ξ, and (c) the surface to volume ratio *S*/*V* with increasing solute volume fraction ϕ_solute_. Note that averaged ϕ_solute_ value (Table 1) was used and the error bar was smaller than the plotted symbol. (d) Scaling behavior of ξ with the square root of the salt concentration (c; in molarity M), which is essentially equivalent to the square root of the [Na^+^]_tot_ in the solutions studied.

The narrowing of the scattering prepeak with increasing concentrations is reflected in an increase in the correlation length ξ (Figure 3b). Note that ξ is inversely proportional to *S*/*V* as shown in eq 5, but the trend in *S*/*V* increases with ϕ_solute_ (Figure 3c). Together, the decrease in *d* and increase in *S*/*V* with ϕ_solute_ (Figure 3) follow the schematic morphology configurations proposed for anion-rich nanodomains (as channels) inside a water-rich matrix presented by Horwitz et al.,^16^ (see their Figure 5). Hence, the behavior of *S*/*V* at high concentrations reflects an increasing size or number of solute-rich nanodomains, both of which would generate an increase in the *S*/*V* ratio with increasing ϕ_solute_. Finally, as ϕ_solute_ nears 0.5, both ξ and *S*/*V* gradually reach stable values (Figure 3b,c). This implies that the factor controlling the solutions morphology at high (enough) concentrations may be geometrical and not a factor of the specific anion (i.e., R2 vs R3 solutions). Similarly, Horwitz et al.,^16^ have shown that, at ϕ_solute_ concentrations greater than 0.5, a constant ξ value and common *S*/*V* trend was observed for both the LiTFSI and LiTf WISEs. As the Tf^–^ anion (a 6 Å diameter sphere) is smaller than the TFSI^–^ anion (an 8 Å long, 3 Å diameter cylinder), the appearance of common trends for both systems suggest that the determining factor in the formation of the WISEs nanostructure is the solute volume fraction (related to the anion size), rather than its molality.^16^

Furthermore, as *a*_0_ = 1/ξ and *q*_0_ = 2π/*d*, in accordance with liquid state theory, the increase in ξ (from ∼2.0 to 4.5 Å) with ϕ_solute_, accompanied by the decrease in *d* (from ∼7.5 to 5.5 Å), indicates that the concentration ranges studied far exceed the Kirkwood transition concentration.^21,48^ Thus, the correlation length ξ represents the underscreening phenomenon at high concentrations, i.e., when the decay (screening) length exceeds the Debye length.^21,48^ The value of ξ observed here (∼4.5 Å in maximum) is of the same order of magnitude as those measured for 1–1, 1–2, and 1–3 chloride salt solutions by SAXS at their highest concentrations, including ξ = 3.9 Å for the 6 m NaCl, 5.7 Å for the 4 m CaCl_2_, 5.2 Å for the 3 m SrCl_2_, and 6.3 Å for the 3 m ErCl_3_ solutions.^19,21^ In 29 m ZnCl_2_ + *x*LiCl bisalt aqueous electrolytes, SANS analysis using the TS model showed that addition of LiCl (up to *x* = 10 m) leads to an increase in ξ from 3.7 to 3.9 and to 6.4 Å at *x* = 0, 5, and 10 m, respectively.^13^ In addition, SANS profiles of the LiTFSI and LiTf WISEs show an increase in ξ with concentration to a final constant value of ∼6–7 Å for ϕ_solute_ > 0.5 (regardless the anion types; that is above ∼7 and ∼12 m in the LiTFSI and LiTf, respectively).^16^

Therefore, the small-angle scattering data in the literature and in this study support the following hypotheses: (i) all aqueous electrolytes, both simple and complex bisalt systems, show a general trend of increasing correlation length (i.e., underscreening length) at high concentrations; and (ii) the correlation lengths are limited to a few to tens of Å before precipitation occurs. The scaling behavior of the correlation length in aqueous electrolytes above the Kirkwood transition, motivated by an experimental work using a surface force balance,^50^ is important from both a fundamental and applied point of view. For example, in high-concentration electrolytes, the underscreening nature helps explain the emergent phenomena that govern bulk and interfacial electrolyte structures. This plays a crucial role in electrochemistry and in the stability of charged (nano)­colloid dispersions for both spherical^51^ and nonspherical colloids at high electrolyte solutions.^52,53^ There are many theoretical predictions on the scaling of the underscreening length based on long- and short-range interactions and different choices of relative permittivity (to compute the Debye length) and of the ion diameter.^50,54,55^ In general, the underscreening length in concentrated electrolytes is expected to scale as ξ ∝ *c* ^(α/2)^, with *c* being molar (M) concentration and the scaling exponent α ranging between 1 and 2 by the theoretical studies.^21,54–56^ As shown in Figure 3d, we obtained scaling exponents α = 1.26 ± 0.51 and 1.25 ± 0.67 for the R2 and R3 solution series, respectively, and values between 1 and 1.5 have been reported from SAXS and SANS measurements,^21,55^ all of which lie within the theoretical range.^21,54–56^ Such small scaling exponent for bulk electrolyte implies that a maximum correlation length at or near its solubility limit would still be in the range of a few to tens of Å (e.g., if an electrolyte has a solubility of 15 M and a scaling exponent α of 2, it implies that the correlation length ξ is roughly 15 Å, given the proportionality prefactor not far from 1).

### Connection to the Molecular Relaxation Processes

QENS, ^1^H NMR, and dielectric relaxation spectroscopy (DRS) have previously been used to investigate picosecond (ps) time scale motions of H-bearing species at two concentrations along the R3 solution line (Figure 1; green circles).^5^ The scattering wavevector range *Q* = 0.3–1.9 Å^–1^ probed by (incoherent) QENS measurements covers the (coherent) SANS prepeak maximum at *Q* of ∼0.85 to 1.11 Å^–1^. The *Q*-dependence of the QENS peak broadening revealed two molecular relaxation processes on 40–60 ps and 350–750 ps time scales at room temperature.^5^ The fast QENS component, in agreement with ^1^H NMR rotational correlation times and the DRS relaxation times, was assigned to local relaxation processes (a minimum lifetime) related to solvated cluster motions, e.g., backbone tumbling of the intact bonds in the clusters or reorganization of local bonding via exchange mechanisms. The slow QENS component is likely due to restricted diffusional dynamics, reflecting collective structural relaxation processes that occur in a densely packed solution. The activation energies of the fast component are ∼35–45% smaller than those of the slow component.^5^ This means that disrupting water–ion percolated networks is harder than breaking bonds (e.g., via water exchanges) in the solvated molecular species. These microscopic dynamics, particularly greater local/confined motions for ionic complexes, agree with the proposed solution morphology based on SANS results, i.e., nonhomogeneous species distributions, solute aggregation, and formation of an interconnecting water–ion network for nanostructural “caging” effects.

## Conclusions

In this work, we studied the effect of anion size/ratio (OD^–^ vs Al­(OD)_4_^–^) and total salt concentration on the nanostructure of alkaline sodium aluminate bisalt electrolytes. From an anion–anion interaction point of view, the migration of the SANS prepeak from *d* ∼ 7.5 to 5.5 Å as the concentration increased suggests the formation of complex anionic molecular networks (potentially involving aluminate polymerization) from isolated aqueous anions. The SANS profiles reflect structural nanoheterogeneities facilitated by D-bonding interactions between water molecules and aluminate species and the participation of deuteroxide ions in both the water-rich and aluminate-rich nanodomains. The short correlation lengths ξ (∼4.5 Å in maximum) reflecting electrostatic underscreening observed at solute volume fractions close to 0.5 (regardless of the anion types) are similar in length to that reported for other simple salt or complex bisalt aqueous electrolytes. This may indicate that the correlation length is generally limited to a few to tens of Å at high concentrations. Furthermore, the comparison between the SANS results and our previous QENS study furnished insights into how structural nanoheterogeneities are associated with microscopic molecular relaxation processes. The fact that the activation energy of the collective structural relaxation processes is greater than that of local molecular transport by bond reorganization implies that the heterogeneity in network structure modifies the microscopic dynamics, leading to the macroscopic behavior observed in the concentrated electrolyte system studied. This includes prolonged metastability, frustrated crystallization behavior (at room temperature), and good glass-forming ability (upon supercooling to ∼200–150 K). This new fundamental insight into concentrated electrolyte structure will inform models to predict their response in industrially relevant systems such as the Bayer process for aluminum production, WISEs for energy storage, and the process of radioactive waste at the U.S. DOE’s legacy nuclear sites.

## Supplementary Material



## Data Availability

The data that support the findings of this study are available from the corresponding author upon request.

## Notes

Descriptions about solution density estimation; weighting factor descriptions in neutron coherent scattering; approximated solution density and solute volume fraction; illustration of SANS data fitting with Teubner–Strey model; neutron scattering weights (as %) of pairwise partial contributions; and the parameters (*d*, ξ, and *S*/*V*) obtained from the fitting (PDF)
