# Supplementary material for: Nanostructuring and Underscreening of Bisalt Electrolytes with Dual-Anion Effects: Insights from Small-Angle Scattering Prepeak Analysis
Source: J Phys Chem B. 2026 Jul 27;130(31):7978–86. doi: 10.1021/acs.jpcb.6c01734 (PMC13455053; doi:10.1021/acs.jpcb.6c01734)
Supplement: Supplementary file 1 [file jp6c01734_si_001.pdf]

Supporting Information for:

Nanostructuring and Underscreening of Bisalt Electrolytes with Dual-Anion Effects: Insights from Small-Angle Scattering Prepeak Analysis

Hsiu-Wen Wang,[†]* Emily T. Nienhuis,[‡] Ashley R. Kennedy,[‡] Sebastian T. Mergelsberg,[‡] Valerie Munoz,[‡] Lawrence M. Anovitz,[†] Gergely Nagy,[&] Jacob G. Reynolds,[§] Jaehun Chun,[‡] Gregory K. Schenter,[‡] Carolyn I. Pearce[‡]*

[†]Chemical Sciences Division and [&]Neutron Scattering Division, Oak Ridge National Laboratory, Oak Ridge, Tennessee 37831, United States

[‡]Pacific Northwest National Laboratory, Richland, Washington, 99352, United States

[§]Washington River Protection Solutions, LLC, Richland, Washington 99352, United States

Corresponding Authors

*Hsiu-Wen Wang: wanghswen@gmail.com

*Carolyn I. Pearce: carolyn.pearce@pnnl.gov

This file contains:

Supporting Text-1 and Text-2

Figures S1 and S2

Tables S1 and S2

Supporting Text-1: Steps for solution density estimation

The density of solutions in this work is estimated using the following steps:

(1) Experimental densities of aqueous NaOH solutions measured at 25 ºC, over the entire concentration range, were obtained from Sipo *et al.*[1] and Clegg *et al.*[2] A hypothetical NaOH melt density of 1.960 g/cm^3 at 25 ºC reported by Clegg *et al.*[2] was used. We adopted the hypothetical melt density value and the measured densities of aqueous NaOH solutions (up to the solubility limit) as a fitted data set, and repeated the model method described by Clegg *et al.*[2] (see their Figure 67) to obtain the calculated density of superstrated aqueous NaOH solutions. For example, calculated density of a 30 m NaOH solution is approximately 1.560 g/cm^3 at 25 ºC, and is consistent (±0.002 g/cm^3) with the value calculated using the empirical Masson equation presented by Sipos *et al.*[3]

(2) Work by Sipos *et al.*[3] also reported densities of hypothetical (pure) $NaAl(OH)_4$ aqueous solutions as a function of ionic strength up to about 10 M at 25 ºC (see their Figure 3 and Table 3). Using their reported densities and the correlated Masson equation, we then calculated density of a hypothetical (pure) $NaAl(OH)_4$ solution at a higher ionic strength concentration. For example, a 30 m pure $NaAl(OH)_4$ aqueous solution corresponds to ionic strength of 11.323 M and density of 1.679 g/cm^3 at 25 ºC.

(3) In line with the description by Sipos *et al.*,[3] if we assume that the $NaAl(OH)_4$-NaOH-H_2O ternary solution system consist only of Na^+, OH^-, $Al(OH)_4^-$, and water molecules, the system can then be regarded as a mixture of pure NaOH and pure $NaAl(OH)_4$ aqueous solutions. The density of such ternary system can consequently be formulated in terms of the densities of the respective binary solutions, i.e., NaOH-H_2O as described in the step-1 and $NaAl(OH)_4$-H_2O as described in the step-2. Using the Young's rule expressed by Sipos *et al.*[3] (their equation (2)), and density values from steps-1 and -2, we then estimated solution density at the studied molality concentrations in this work. For solutions at lower concentrations, such as $[Na^+]_{tot}$ = ~5 or 10 m (in either R2 or R3 series), we also performed a simple crossed check (based on solution's average molecular weight) with densities reported by Radnai *et al.*[4] and Li *et al.*[5] Note that up to this point, all the calculations are for the protiated (H) scenario.

(4) The last step is to consider atomic weight differences between H and D atoms. For isotope substitutions, replacing H for D only changs the mass but not the volume of the solution. Thus, the estimated density from the step-3 simply scales with the solution mass ratio between

deuterated (D) vs. protiated (H) scenarios. Results for the estimated solution densities at 25 ºC are summarized in Table 1 of the main text, and are shown in Figure S1a, together with the approximated V_{solute} (cm^3/mole) and ϕ_{solute} values (using estimated densities) as a function of the $[Na^+]_{tot}$ concentrations.

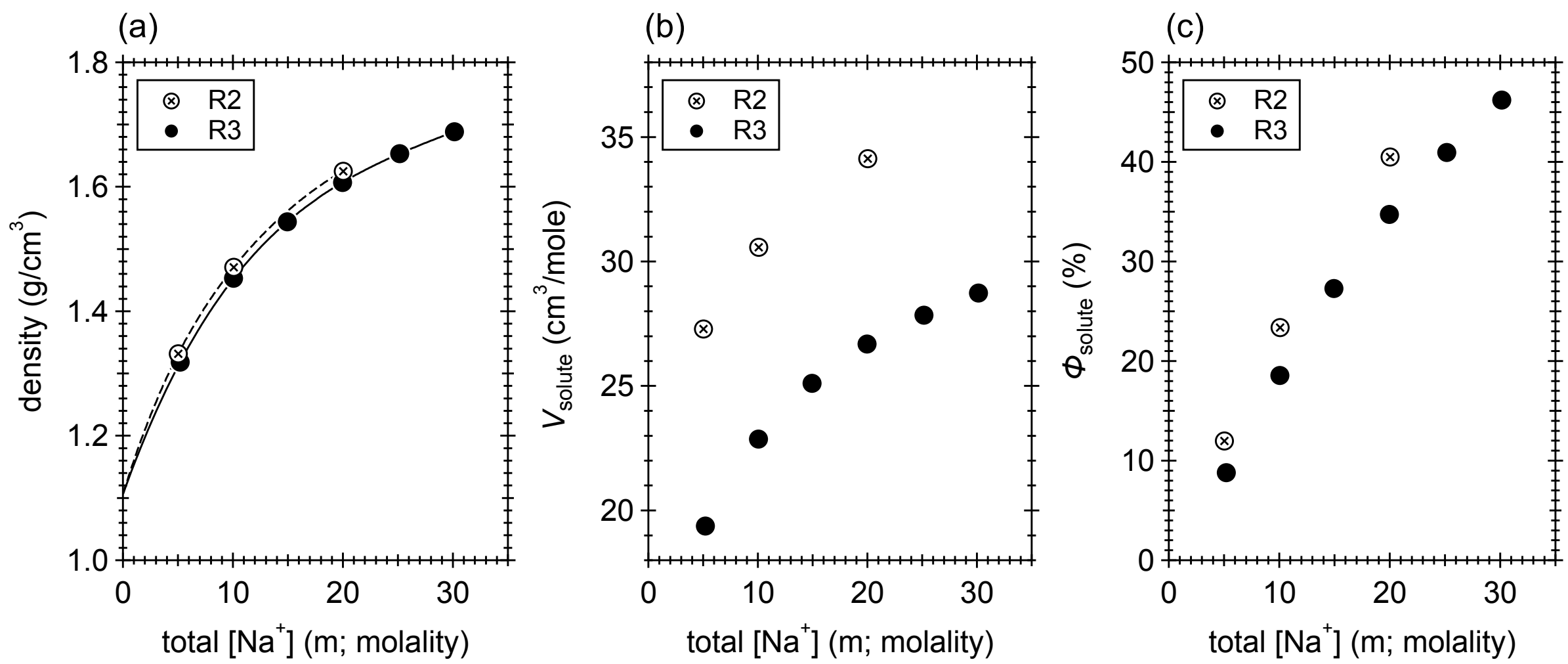

Figure S1. Approximated (a) solution density, (b) apparent molar volume of the solute phase, and (c) solute volume fraction of the deuterated alkaline sodium aluminate electrolytes studied by this work.

Supporting Text-2: Pair-wise weighting factors in neutron coherent scattering cross section

The normalized weighting factor for neutron scattering is calculated as:

$$w_{ij} = \frac{A_{ij}}{\sum A_{ij}} = \frac{(2-\delta_{ij})c_i c_j b_i b_j}{\sum_i (c_i b_i)^2} \qquad (1)$$

where δ_{ij} is the Kronecker delta, c_i (or c_j) is the stoichiometric fraction of element *i* (or *j*), b_i (or b_j) is the neutron coherent scattering length (fm = 10^{-15} m) of element *i* (or *j*). The $\sum_i (c_i b_i)^2 = \sigma_c$ is the microscopic coherent scattering cross section (typically in unit of barns/atom, where 1 barn = 10^{-24} cm^2). Mathematically, there are *s*(*s*+1)/2 partial pair-wise contributions in a system containing *s* species (*s* = atoms or molecules). Thus, in the deuterated alkaline sodium aluminate bisalt electrolyte ($NaAl(OD)_4$- NaOD-D_2O), a single scattering data contains a total of 10 partial pair-wise contributions if one do not differentiate oxygen and deuterium sites in D_2O, OD^- and aluminate molecules. These 10 partials are: Na-Na, Na-O, Na-D, Na-Al, O-O, O-D, D-D, Al-Al, Al-O, and Al-D. Alternatively, one can make the distinction among O_w/O_d/O_a sites and D_w/D_d/D_a sites (for oxygen and deuterium sites in D_2O, OD^- and aluminate molecules, respectively) in the

weighting factor calculation to facilitate interpretation of their relative contributions in the corresponding experimental data. In fact, this differentiation among different oxygen and deuterium sites provides a tool to justify molecular species contributions, especially after grouping partial atomic pair-wise correlations into a sub-ensemble representation of a molecular species of interest. For example, water-water correlations in a salt solution, including both intra- and intermolecular terms, can be expressed by the sum of the three atomic pair-wise partials: $\Sigma A_{wat.} = A_{\mathrm{D}_w\mathrm{D}_w} + A_{\mathrm{D}_w\mathrm{O}_w} + A_{\mathrm{O}_w\mathrm{O}_w}$. In a similar fashion, aluminate-aluminate correlations comprise the sum of six partials: $\Sigma A_{\mathrm{alum.}} = A_{AlAl} + A_{AlO_a} + A_{AlD_a} + A_{O_a\mathrm{O_a}} + A_{\mathrm{O_aD_a}} + A_{\mathrm{D_aD_a}}$. Aluminate-water intermolecular correlations, on the other hand, comprises another sets of six partials: $\Sigma A_{\mathrm{alum.\text{-}wat.}} = A_{\mathrm{AlD_w}} + A_{\mathrm{AlO_w}} + A_{\mathrm{O_aD_w}} + A_{\mathrm{O_aO_w}} + A_{\mathrm{D_aD_w}} + A_{\mathrm{D_aO_w}}$.

Table S1 summarized the calculated neutron scattering weights (as % to the total observed intensity) of all the pair-wise contributions in the solutions studied. For simplicity, only the aluminate monomer speciation is assumed, i.e., all Al^{3+} ions are presented as the $Al(OD)_4^-$ monomer. For other speciation cases, such as in terms of the di-μ_2-deuteroxo $Al_2(OD)_8^{2-}$ or the μ_2-oxo $Al_2O(OD)_6^{2-}$ dimers, one can refer to our previous neutron total scattering work.[6] The stoichiometric fractions c_i are converted from the solution compositions listed in Table 1 of the main text. For example, if all Al^{3+} ions are in the form of $Al(OD)_4^-$, the 20.032 m $[Na^+]_{tot}$ and 9.993 m $[Al^{3+}]_{tot}$ solution in the R2 series has speciation mole numbers as: 9.993 $Al(OD)_4^-$ + 20.032 Na^+ + 10.039 $OD^-_{(excess)}$ + 55.506 D_2O (note: the excess OD^- ions are needed to balance charges). Note that the weighting factors for the OD^-_{ex}-D_2O, aluminate-D_2O, and D_2O-D_2O partials (no. 6, 9 and10 in Table S1) contribute up to about 73-95% of the total measured intensity, depending on the solution compositions. The scattering weights containing Na^+ correlation partials (no. 1-4 in Table S1), by comparison, contribute weakly to the total intensity. This is because the neutron coherent scattering length for Na (3.63 fm) and Al (3.449 fm) atoms is smaller than O (5.805 fm) and D (6.671 fm) atoms. The prevalent O and D atoms in the current study solutions and the O- and D-containing solute or solvent species are, thus, expected to be the dominated contributors to the measured intensities.

Values listed in Table S1 should not be confused with the scattering length densities (e.g., ρ_{solute} or ρ_{solvent}) and the scattering length density contrast (e.g., $\Delta\rho^2 = (\rho_{\mathrm{solute}} - \rho_{\mathrm{solvent}})^2$). The scattering length density (not the scattering length) is crucial for calculating contrast in neutron scattering experiments (small-angle or reflectivity), and is defined as the ratio of the

scattering length per molecule ($\sum b_i$; 10^{-15} m = 10^{-13} cm) and the molecular volume (V_m; cm^3). For example. assuming a $NaAl(OD)_4$ molecule, the scattering length density is given as:

$$\rho_{\mathrm{NaAl(OD)_4}} = \frac{\sum b_i}{V_m} = \frac{(b_{Na} + b_{Al} + 4b_O + 4b_D)}{V_m} \qquad (2)$$

where V_m is usually computed from the known bulk density (*d*; g/cm^3) of the molecule and the molecular weight (*M*; g/mole) as:

$$V_m = \frac{M}{dN_a} \qquad (3)$$

where N_a is Avogradro's number (6.02×10^{23}/mole). Note that the scattering length density has unit of cm^{-2} (or Å^{-2}). In aqueous salt solutions with solute clustering phenomena, knowledge about ion participation between the water-rich and solute-rich phase domains typically requires insights from advanced molecular simulations. Without this information, we do not know what the appropriate "molecular composition" and "molecular volume" should be, and hence the scattering length density of the solute-rich phase (ρ_{solute}) or water-rich phase (ρ_{solvent}) and their contrast factor ($\Delta\rho^2$) cannot be calculated. Nonetheless, given the (atomic) number density N/V (number of scattering nuclei/cm^3) in a material, a macroscopic coherent scattering cross section is defined as: $\Sigma_c = (N/V)\,\sigma_c$ (units of cm^{-1}). SANS data are often presented on an absolute macroscopic cross section and are scaled independent of instrumental conditions and of sample volume. Thus, the measured SANS $I(Q)$ intensity is proportional to the macroscopic coherent scattering cross section as: $I(Q) \propto (d\Sigma_c/d\Omega) \propto (N/V)\,(d\sigma_c/d\Omega)$, where $d\Omega$ is a scattering solid angle. As expressed by equation (2) in the main text, the SANS $I(Q)$ is the product of many factors, but the relation of $I(Q) \propto (d\Sigma_c/d\Omega) \propto \left(w_{ij}\sigma_c\right) \propto \Delta\rho^2$ remains valid.

Table S1. Neutron scattering weights (as %) of pair-wise partial contributions for the solutions studied.

	**R2 series with $[Na^+]_{tot}$ (m):**			**R3 series with $[Na^+]_{tot}$ (m):**					
**Pairs**	**5.022**	**10.062**	**20.032**	**5.192**	**10.043**	**14.936**	**19.960**	**25.159**	**30.133**
1: Na-Na	0.02	0.07	0.16	0.02	0.07	0.13	0.19	0.25	0.31
2: Na-OD_{ex}	0.07	0.22	0.57	0.11	0.33	0.59	0.88	1.17	1.43
3: Na-D_2O	2.50	3.80	4.80	2.70	4.18	5.07	5.61	5.92	6.06
4: Na-Alum.	0.31	0.96	2.41	0.24	0.70	1.27	1.88	2.49	3.06
5: OD_{ex}-Alum.	0.54	1.65	4.15	0.54	1.61	2.91	4.30	5.71	7.00
6: OD_{ex}-D_2O	4.30	6.54	8.27	6.19	9.58	11.63	12.86	13.56	13.88
7: OD_{ex}-OD_{ex}	0.06	0.19	0.49	0.13	0.38	0.68	1.00	1.33	1.64
8: Alum.-Alum.	1.15	3.51	8.83	0.58	1.72	3.11	4.60	6.11	7.49
9: Alum.-D_2O	18.30	27.84	35.22	13.26	20.49	24.87	27.52	29.01	29.70
10: D_2O-D_2O	72.75	55.23	35.10	76.24	60.94	49.74	41.17	34.44	29.43

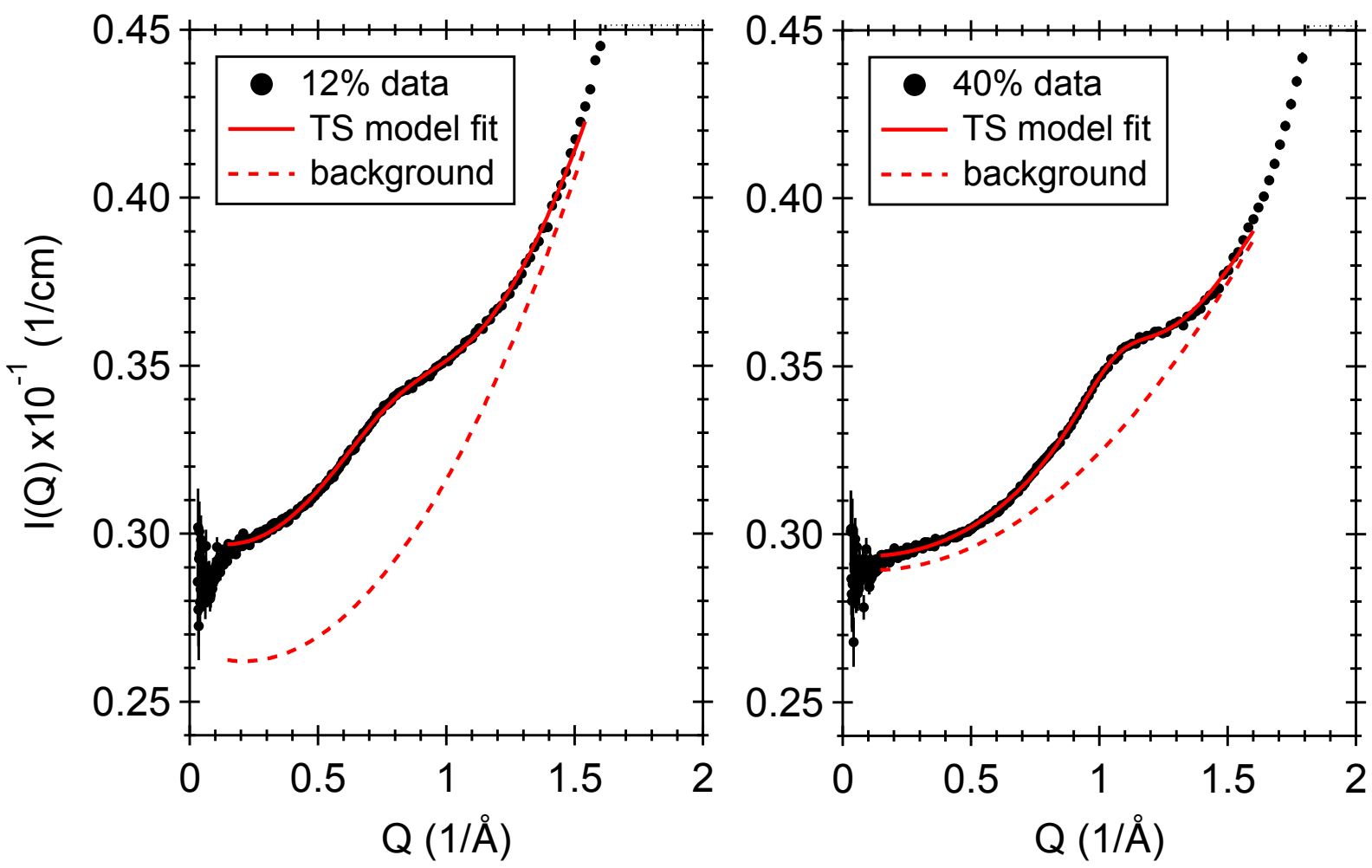

Figure S2. Illustration of small-angle neutron scattering data fitting with Teubner-Strey model. In the model fit (on the Q range from 0.16 to 1.6 Å^{-1}), the intensity is calculated as: $I(Q) = \frac{1}{a_2+c_1Q^2+c_2Q^4} + (k_2Q^2 + k_1Q + k_0)$, where parameter a_2 acts as a scale factor, and since it is in the denominator, it also influences the magnitude of c_1 and c_2 parameters. The background was performed with a quadratic polynomial function $(k_2Q^2 + k_1Q + k_0)$. The second-order term for the background is needed, due to the proximity of the prepeak to the primary peak at a larger region of high Q values.

Table S2. Parameters d, ξ, and S/V from the fitting (e.s.d. values in parentheses).

	**[Na$^+$]$_{tot}$ (m)**	$\boldsymbol{\phi}_{\mathbf{solute}}$	$\boldsymbol{d}$ **(Å)**	$\boldsymbol{\xi}$ **(Å)**	$\boldsymbol{S/V}$ **(1/ Å)**
**R2**	5.022	0.12	7.38(47)	2.22(57)	0.19(5)
	10.062	0.23	6.74(20)	3.07(80)	0.23(6)
	20.032	0.40	5.80(13)	4.19(133)	0.23(7)
**R3**	5.192	0.09	7.21(57)	2.10(64)	0.15(5)
	10.043	0.19	6.43(19)	2.74(62)	0.22(5)
	14.936	0.27	6.02(14)	3.31(92)	0.24(7)
	19.960	0.35	5.68(14)	4.39(169)	0.21(8)
	25.159	0.41	--	--	--
	30.133	0.46	--	--	--

References

(1) Sipos, P. M.; Hefter, G.; May, P. M. Viscosities and densities of highly concentrated aqueous MOH solutions (M^+= Na^+, K^+, Li^+, Cs^+,$(CH_3)_4N^+$) at 25.0 °C. *J. Chem. Eng. Data* **2000**, *45*, 613–617.

(2) Clegg, S. L.; Wexler, A. S. Densities and apparent molar volumes of atmospherically important electrolyte solutions. 1. The solutes H_2SO_4, HNO_3, HCl, Na_2SO_4, $NaNO_3$, NaCl, $(NH_4)_2SO_4$, NH_4NO_3, and NH_4Cl from 0 to 50 °C, including extrapolations to very low

temperature and to the pure liquid state, and $NaHSO_4$, NaOH, and NH_3 at 25 °C. *J. Phys. Chem. A* **2011**, *115*, 3393–3460.
(3) Sipos, P.; Stanley, A.; Bevis, S.; Hefter, G.; May, P. M. Viscosities and densities of concentrated aqueous NaOH/$NaAl(OH)_4$ mixtures at 25 °C. *J. Chem. Eng. Data* **2001**, *46*, 657–661.
(4) Radnai, T.; May, P. M.; Hefter, G. T.; Sipos, P. Structure of aqueous sodium aluminate solutions: a solution X-ray diffraction study. *J. Phys. Chem. A* **1998**, *102*, 7841–7850.
(5) Li, J.; Prestidge, C. A.; Addai-Mensah, J. Viscosity, density, and refractive index of aqueous sodium and potassium aluminate solutions. *J. Chem. Eng. Data* **2000**, *45*, 665–671.
(6) Nienhuis, E. T.; Pouvreau, M.; Graham, T. R.; Prange, M. P.; Page, K.; Loring, J. S.; Stack, A. G.; Clark, A. E.; Schenter, G. K.; Rosso, K. M.; Pearce, C. I.; Wang, H.-W. Structure and reactivity of sodium aluminate complexes in alkaline solutions. *J. Mol. Liq.* **2022**, *367*, 120379.
